# Effect of experimental infectious bursal disease virus on clinical signs and pathogenesis of avian influenza virus H_9_N_2_ in turkey by real time PCR

**DOI:** 10.30466/vrf.2018.75860.2013

**Published:** 2019-12-15

**Authors:** Farhad Hashemzade, Mansour Mayahi, Abdolhamdi Shoshtary, Masoud Reza Seyfi Abad Shapouri, Masoud Ghorbanpoor

**Affiliations:** 1 *DVSc Graduate, Faculty of Veterinary Medicine, Shahid Chamran University of Ahvaz, and Chief of Poultry Diseases Specialized Hospital, Khoy, Iran* *;*; 2 *Department of Clinical Sciences, Faculty of Veterinary Medicine, Shahid Chamran University of Ahvaz, Ahvaz, Iran; *; 3 *Department of Avian Diseases, Razi Vaccine and serum research institute, Karaj, Iran; *; 4 *Department of Pathobiology, Faculty of Veterinary Medicine, Shahid Chamran University of Ahvaz, Ahvaz, Iran* *.*

**Keywords:** Avian influenza, Infectious bursal disease virus, Real time PCR, Turkey

## Abstract

Infectious bursal disease virus (IBDV) in turkeys may result in immunosuppression, and inability of turkeys to resist nonpathogenic or less pathogenic organisms. A total number of 120 day-old commercial male turkeys were purchased and blood samples were collected from 20 day-old turkeys, remaining 100 were divided into four equal groups and kept in separated rooms. Groups 1 and 2 were infected with 10^4 ^CID_50 _of IBDV via intra-bursal route on day 1; Groups 1 and 3 were each infected with 10^6^ EID50 of AIV (H_9_N_2_) via the oculo-nasal routes on day 30. All groups were vaccinated against Newcastle disease vaccine (NDV). Detection of avian influenza virus H_9_N_2_ in trachea and cloaca swabs and in the tissues, was confirmed by Real-time polymerase chain reaction. Anti- NDV–AIV and anti-IBD titers were measured using HI and ELISA tests, respectively. The present study showed that infectious bursal disease changed the pathogenesis of (AIV) H_9_N_2_ by affecting AI virus replication and resulted in an increase shedding due to prolonged duration of sever clinical signs. The extent of shedding and virus replication need further study.

## Introduction

Avian influenza viruses (AIV) were first recognized in the mid-20^th^ century.^1^^,^^2^ Avian influenza virus H_9_N_2_ is non-pathogenic and has been recognized in various domestic poultry species that is less severe than HPAI.^3^^,^^4^ Avian influenza H_9_N_2_ virus was isolated during an outbreak in chickens in Iran. ^5^ In Europe, the H_9_N_2_ subtype has been detected sporadically in wild birds and poultry; however, in recent years, a number of outbreaks in turkey flocks were reported in Germany, Italy, England, and Poland.^6^ The Avian influenza H_9_N_2_ subtype in Poland was identified in fattening turkey flocks and the clinical signs included drop in feed and water intake, depression, respiratory signs and mortality.^7^ Turkey with less than four weeks of age challenged by secondary pathogens in the 1999 Italian H7N1 LPAI with mortality rates as high as 97.00% has been reported.^8^ Historically, In breeder turkeys in Minnesota (USA) LPAI prevalence took place in the fall during the 1970s and 1980s.^9^^,^^10^ Avian influenza subtypes H1N1, H1N2, or H3N2 and other subtypes of swine-origin influenza viruses, either by mechanical ways or via humans infected with swine-origin influenza viruses can infect turkeys.^11^ Infectious bursal disease (IBD) is an acute, highly contagious viral infection of young chickens and bursa of Fabricius is main virus target. The most important manifestation is severe, prolonged immuno-suppression in the chickens infected in early age. The outcome of the immunosuppression caused by the IBD viruses predisposes birds to some other infections, including gangrenous dermatitis, inclusion body hepatitis-anemia syndrome, *Escherichia coli* infections and vaccination failures. The maintenance of young chicks from the early stages of IBD virus infection is critical.^12^^,^^13^ In turkeys, classical virulent IBDV strains produces only subclinical forms of the disease. Very virulent IBDV (vvIBDV) isolate from the bursa of turkey and Its identity have been recognized by RT-PCR and restriction analysis of the product.^13^ In Nigeria four turkey flocks with clinical symptoms of IBD was distinguished. The turkey isolates were recognized within two of the three VV-clusters of chicken isolates. Close relation of a turkey isolate (NIE009t) to vvIBDV strain D6948NET for both segment A (1.40% sequence diversity) and segment B (2.10%) has been recognized by full length sequence.^14^^,^^15^ The present study was undertaken to evaluate the effects of experimental infection of IBDV on pathogenesis of avian influenza virus H_9_N_2_ in turkey by real time PCR and evaluation of humoral immunity system. 

## Materials and Methods

**Viruses. **AIV H_9_N_2_ (A/Chicken/Iran/688/1999) and IBDV Cloned, IR499 (accession number: EU09153) were obtained from Razi Vaccine and Serum Research Institute (Karaj, Iran). The AIV was propagated two times in 9 to 11-day-old embryonated chicken eggs and IBDV was propagated in negative IBDV antibody chicken. The embryo infective dose (EID_50_) and the chicken infective dose (CID_50_), for AIV and IBDV were respectively calculated according to the formula of Reed and Muench.^16^

**Experiment plan. **Research plan designed according to animal welfare ethics (EE/98.24.3.38672/scu.ac.ir). 

A total number of 120 day-old commercial male turkeys (strain converter hybrid France) were purchased and blood samples were collected from 20 day-old turkeys, remaining 100 were divided into four equal groups. Birds were reared in separate rooms in the Poultry Research Unit, Faculty of Veterinary Medicine in Ahvaz and received feed and water *ad libitum* during the experimental period. The turkeys’ room temperature started from 38.00 ˚C and weekly decreased 3.00 ˚C up to 21.00 ˚C and remained stable during experiment The All turkeys were fed pelleted feed composed of corn, soybean, dicalcium phosphate, carbonate calcium premix vitamin, minerals, and balanced crude protein and energy depend on the ages, however, coccidiostats and antimicrobials were not used. Chicks in Groups 1 and 2 were infected with 10^4^ CID_50 _of IBDV via intrabursal (IB) route on day 1of age. ^17^ Groups 1 and 3 were infected with 10^6^ EID_50 _of AIV (H_9_N_2_) via the oculo-nasal routes on day 30. Blood samples were collected from 10 chicks of each group via the wing vein on days 30, 37, 44, 51 and 58 to determine AIV antibodies using HI test.^11^ The ELISA test was performed to detect and assay the IBDV antibody in serums of 1, 35 and 58 day old chicks using MPR4 kit (IDEXX, Regensburg, Germany). Three turkeys from each experimental group were randomly collected at 3, 7, 11 and 15 days post AIV challenge, and euthanized by intravenous injection sodium pentobarbital (50.00 mg kg^-1^) and tracheas, feces, lungs and kidney samples were collected.

**RNA isolation. **All samples were immediately stored at – 70.00 ˚C until used. Thereafter, all tissue samples collected were homogenized with triptose phosphate buffer and centrifuged for 5 min. Then, the supernatant liquid was stored at – 70.00 ˚C until required. RNA was extracted from the samples using a high pure viral nucleic acid kit (Roche Applied Science, Mannheim, Germany) according to the manufacturer’s instructions. Briefly, 1.00 mL of RNX solution was added to 100 μL of each sample of homogenized tissue. After addition of 200 μL chloroform, the mixture was centrifuged at 12,000 rpm at 4.00 ˚C for 15 min. The upper phase was added to an equal volume of isopropanol and centrifuged at 12,000 rpm at 4.00 ˚C for 15 min. After the washing step, the pellet was dissolved in a final volume of 50.00 μL distilled water (DW).^18^

**Real time PCR. **The reaction was performed with a mixture of 20.00 pmol random hexamer and 20.00 pmol of primer which was specific to a highly conserved region of matrix protein gene of influenza A virus, previously described by Lee *et al*. and Dohms *et al*.^19^^,^^20^ The reaction mixture was incubated at 50.00 ˚C for 30 min for the production of cDNA and then incubated at 95.00 ˚C for 15 min and 45 cycle. Each cycle contained: 1) denaturization: 94.00 ˚C for 45 sec and 2) synthesis: 60.00 ˚C for 45 sec using Rotor-Gene 3000 (Corbett Research, Sydney, Australia). Primers and probe.^21,22 ^Forward H9: 5'-ATGGGG TTTGCTGCC-3', Reverse H9: 5'-TTATATACAAATGTTGCAC (T)CTG-3', and Probe H9: 5'-TTCTGGGCCATGTCCAATGG-3'.

**Statistical analyses. **The mean titer of chickens’ AIV virus response was evaluated by independent T – samples *t*-test, and HI test was evaluated by one-way ANOVA, with statistical comparisons allowed among the different groups using SPSS (version 19.0; IBM, Chicago, USA). Qualitative real time PCR were quantification by quantity DNA primary model and quantity cycle threshold (Ct) according to formula:

*Y= mX + b*

and quantity of AI (H_9_N_2_) were calculated according to EID_50 _per 100 µL, where, Y=Ct, X= log_10_ sample quantity, m = slope equal to – 3.75 and b = width from origin equal to 39.06.

## Results

The AIV (H_9_N_2_) was detected in the trachea on days 3, 7 and 11 days post inoculation (DPI) in only groups 1 and 3 but not in the 2^nd^ and 4^th^ (control) groups (data zero). As shown in Table 1 the amount of AIV in the trachea on days 3 and 7 DPI in Group 1 was higher than in group 3. As shown in Table 2, the amount of AIV detected in the feces on days 7 and 11 DPI was higher than group 1. The amount of AIV in group 1 on day 7 was higher than group 3. As shown in Table 3, AIV was detected on days 3, 7 and 11 in the lungs of groups 1, 11 in the lungs of groups 1 and 3, however, its amount in group 1 was higher than in group 3, and the virus was not detected on day 15 in all groups. However, the most pronounced detection of AIV was in the kidneys, as shown in Table 4. AIV was detected in the kidneys on days 3, 7, 11 and 15 of groups 1 and 3, with the lowest level being seen in this organ. As shown in Table 5, there was an increase in the antibody titre against AI virus H_9_N_2_ on day 30 in groups 1 and 3, and the highest titres were observed on day 14 after inoculation. The antibody titres against AI virus H_9_N_2_ reached 2^-6.9^ (log_2_) in group 3 and reached 2^-4.7^ (log_2_) in group 1. Table 5 shows that antibodies against IBD virus in group 1 on days 35 and 58 were 2961 and 2016, respectively, and group 2 were 2749 and 2046, respectively, and other groups were negative.

**Table 1:** The amount of AIV (log_10_) in tracheal samples in different days post inoculation (DPI) with AIV

**Groups**	**3 DPI **	**7 DPI **	**11 DPI **
**1**	4.08 ± 0.15^a^	4.96 ± 0.11^a^	1.64 ± 0.19^a^
**2**	ND^b^	ND^b^	ND^b^
**3**	2.12 ± 0.13^c^	4.03 ± 0.07^c^	1.56 ± 0.09^a^
**4**	ND^b^	ND^b^	ND^b^

ND: Not detected.

^abc^ Different superscript letters in each column indicate significant difference between mean virus amount (*p *< 0.05).

**Table 2:** Amount of AIV (log_10_) in fecal samples in different days post inoculation (DPI) with AIV

**Groups**	**3 DPI**	**7 DPI**	**11 DPI**
**1**	1.47 ± 0.80^a^	3.82 ± 1.20^a^	3.04 ± 0.95^a^
**2**	ND^b^	ND^b^	ND^b^
**3**	1.86 ± 0.80^a^	2.50 ± 0.34^a^	2.34 ± 0.04^a^
**4**	ND^b^	ND^b^	ND^b^

ND: Not detected.

^ab^ Different superscript letters in each column indicate significant difference between mean virus amount (*p *< 0.05).

**Table 3:** Amount of AIV (log_10_) in lung sample in different days post inoculation (DPI) with AIV

**Groups**	**3 DPI **	**7 DPI **	**11 DPI **	**15DPI **
**1**	3.84 ± 0.22^a^	3.71 ± 0.12^a^	2.83 ± 0.05^a^	0.92 ± 0.13^a^
**2**	ND^ b^	ND^ b^	ND^ b^	ND^ b^
**3**	1.95 ± 0.14^c^	2.15 ± 0.07^c^	1.53 ± 0.09^c^	0.82 ± 0.04^a^
**4**	ND^ b^	ND^ b^	ND^ b^	ND^ b^

ND: Not detected.

^abc^ Different superscript letters in each column indicate significant difference between mean virus amount (*p *< 0.05).

**Table 4:** Amount of AIV (log_10_) in lung sample in different days post inoculation (DPI) with AIV

**Groups**	**3 DPI **	**7 DPI**	**11 DPI **	**15 DPI **
**1**	2.50 ± 0.10^a^	2.3 ± 0.14^a^	2.03 ± 0.15^a^	1.55 ± 0.07^a^
**2**	ND^b^	ND^b^	ND^b^	ND^b^
**3**	0.95 ± 0.07^a^	2.03 ± 0.09^a^	2.55± 0.07^a^	2.32 ± 0.10^a^
**4**	ND^b^	ND^b^	ND^b^	ND^b^

ND: Not detected.

^ab^ Different superscript letters in each column indicate significant difference between mean virus amount (*p *< 0.05).

**Table 5:** Mean titers of AIV (H_9_N_2_) antibody after challenge in day-old turkeys by IBDV and 30-day olds by AIV

**Groups**	**Day 30**	**Day 37**	**Day 44**	**Day 51**	**Day 58**
**1**	1.30 ± 0.15^a^	3.20 ± 0.21^a^	4.70 ± 0.19^a^	4.35 ± 0.15^a^	4.25 ± 0.30^a^
**2**	1.50 ± 0.17^a^	0.90 ± 0.18^b^	1.10 ± 0.1^b^	0.80 ± 0.13^b^	0.40 ± 0.18^b^
**3**	1.40 ± 0.16^a^	3.20 ± 0.11^a^	6.90 ± 0.18^c^	6.05 ± 0.19^c^	5.70 ± 0.25^c^
**4**	1.40 ± 0.16^a^	1.30 ± 0.13^b^	1.20 ± 0.13^b^	0.70 ± 0.15^b^	0.60 ± 0.16^b^

^abc^ Different superscript letters in each column indicate significant difference between mean virus amount (*p *< 0.05).

## Discussion

Over the years, more and more real-time PCR has been utilized for the detection of pathogenic viruses because of its rapidity, simplicity, sensitivity, specificity and ability to quantify infection levels.^21^ To study the pathogenesis of H_9_N_2_ low pathogenic avian influenza virus, a virus which is responsible for most diseases in domestic poultry in Iran, and the ability of this virus to proliferate in different organs of broiler chickens, TaqMan real-time quantitative PCR assay was used.^23^ The LPAI viruses cause infections which are restricted to the respiratory and gastrointestinal (GI) tracts of chickens.^24^ The detection of the virus in the trachea, lungs and kidneys indicates that H_9_N_2_ AI virus is pneumotropic and nephrotropic, following intranasal inoculation. Viral RNA was not present in all samples before the inoculation of AIV. Predominant infection was observed in the respiratory, tracheal and lung, tract on days 3 and 7 DPI with AIV. In this study, it was demonstrated that the highest frequency of viral RNA detection in the trachea was observed at 3 and 7 DPI in Groups 1 and 7 DPI in Group 3. A comparison of AIV RNA levels at 3 and 7 showed increasing titre of virus in the trachea 7 DPI. A comparison of the mean amount of viral RNA copy of AIV in Group 1 with Group 3, indicated that the viral RNA copy of AIV in Group 1 was very high and with a significant difference. This was responsible for the immunity system suppression by inoculation of IBDV in day-old chicks. The virus was not detected at 11 DPI. The IBDV infection of 1– to 5-day-old turkeys caused a drastic reduction in the plasma cell content of the Harderian gland which lasted for up to seven weeks.^20^ The humoral immune systems of day-old turkeys were repressed by IBDV infection. The effects were differed with virus strain.^25^ The IBDV infected chickens (IBDV+AIV+) shed AI virus for a longer period than the AIV infected birds (AIV+), from both the trachea and cloaca,^26^ which is in accordance with the current study. The AIV H_9_N_2_ was detected in fecal samples in 7 and 11 DPI in Groups 1 and 3. The frequency of viral RNA in Group 1 in 7 and 11 DPI was very high in comparison with Group 3. The highest frequency of viral RNA in the fecal samples were observed on day 7 DPI in Groups 1 and 3. The virus was detected in faeces only on day 6 post inoculation which was in agreement with the current study.^25^ Kwon *et al*. discovered H_9_N_2_ antigen in cloacal swabs following inoculation on days 5 and 7 DPI, which agreed with the current study.^27^ The presence of virus in feces might have resulted from a replication of the virus in the GI tract. High AIV RNA levels in feces at 6 DPI demonstrated high replication of AIV in the GI tract. The highest detection of viral RNA copy of AIV in the lung was observed at 3, 7 and 11 DPI with AIV in Group 1 and 7 DPI with AIV in Group 3. The frequency of viral RNA in Group 1, in 3,7 and 11 DPI, was very high in comparison with Group 3 and this indicated immunity system suppression which wss consequent effect of IBDV. 

A comparison of the amounts of AIV RNA at 3 and 7 DPI with AIV showed that the virus amount in the trachea and lung at 7 DPI was increased but later decreased. The virus was not detected at 15 DPI. Virus was detected in the lungs and trachea from 2 to 4 DPI and this was in agreement with the current study.^18^ The AIV H_9_N_2_ were detected in kidneys on days 3, 7, 11 and 15 DPI in Groups1 and 7, while 11 and 15 DPI were detected in Group 3. In the urinary tract, the predominant infection was observed between days 3 and 9 PI.^23^ The frequency of virus recovery was generally higher for kidney tissues. All kidneys sampled on 1 DPI lacked the viral RNA but viral RNA was identified on 2, 3, 6 and 9 DPI. These data indicated that H_9_N_2_ was nephrotropic.^18^^,^^22^^,^^28^^,^^29^

Presumably, the presence of the virus in the kidneys was resulted from a localized infection of the respiratory tract. The respiratory tract allows the contact and transmission of infectious agents from outside the body into the coelomic cavity.^30^ Mosleh *et al*. detected AIV in the kidneys of inoculated chickens on 3 (40.00%), 6 (60.00%) and 9 (100%) DPI.^23^ This was in agreement with the current study. A comparison of the detection of the virus from the kidneys and the other organs showed that the virus were detected from the kidneys for a longer time. It seems that it would be necessary to continue the sample collection from the kidney for a longer period of time to evaluate the persistence of the virus in the organ. Nucleoproteins of A/Chicken/Iran/259/1998 (H_9_N_2_) isolate was discovered in the kidney and pancreas of 5-week-old chickens after intravenous (IV) inoculation, using an immuno-histochemical technique.^31^ The virus was found only on 8 DPI in kidneys.^24^ In the present study, viral RNA was not present in samples of pancreas obtained from inoculated turkey while nucleoproteins of A/Chicken/ Iran/259/1998 (H_9_N_2_) isolate was detected in the pancreas of 5-week-old after IV inoculation and this was in agreement with the findings of this study.^32^ As shown in Table 5, the antibody titer was increased after inoculation on the day 30 in Groups 1and 3, and the highest titres were observed 14 DPI. The antibody titers reached 2^-6.9^(log_2_) in group 3 and reached 2^-4.7 ^(log_2_) in Group 1. In the comparison with the mean titres of Group 3 and 1 in all samples, it was shown that the mean titres of Group 3 was higher than that of Group 1, and on day 14 this difference was statistically significant. Faragher *et al*. first discovered that IBDV infections had immuno-suppressive effects.^26^ The repression of the produced antibody against Newcastle disease virus was greatly observed in chicks infected on day 1 of age.^26^ Dohms *et al*. demonstrated that IBDV infection of 1– to 5-day-old chicks caused a drastic depletion in plasma cell content of the Harderian gland which lasted for up to seven weeks, although, there are similar contemplations with respect to IBDV infections of turkeys.^20^

It was concluded that infectious bursal disease changed the pathogenesis of AIV (H_9_N_2_) and influenza virus replication, while shedding in infected birds was increased which resulted in prolonged severity and duration of clinical signs. The extent of shedding and virus replication need further study. 

## References

[1] Capua L, Alexander DJ (2004). Avian influenza: Recent developments. Avian Pathol.

[2] Knipe DM, Howely PM (2007). Fields virology. 5th ed. Philadelphia, USA: Williams & Wilkins.

[3] Tong S, Li Y, Rivailler P (2012). A distinct lineage of influenza A virus from bats. Proc Natl Acad Sci USA.

[4] Nili H, Asasi K (2002). Natural cases and an experimental study of H9N2 avian influenza in commercial broiler chickens of Iran. Avian Pathol.

[5] Vasfi Marandi M, Bozorgmehri Fard MH (2002). Isolation of H9N2 subtype of Avian Influenza Viruses during an outbreak in chickens in Iran. Iran Biomed J.

[6] Swieton E, Jozwiak M, Minta Z (2018). Genetic characterization of H9N2 avian influenza viruses isolated from poultry in Poland during 2013/2014. Virus Genes.

[7] Smietanka K, Minta Z, Swieton E (2014). Avian influenza H9N2 subtype in Poland – characterization of the isolates and evidence of concomitant infections. Avian Pathol.

[8] Capua I, Mutinelli F, Marangon S (2000). H7N1 avian influenza in Italy (1999 to 2000) in intensively reared chickens and turkeys. Avian Pathol.

[9] Halvorson DA, Kelleher CJ, Senne DA (1985). Epizootiology of avian influenza: Effect of season on incidence in sentinel ducks and domestic turkeys in Minnesota. Appl Environ Microbiol.

[10] Halvorson DA (2002). Twenty-five years of avian influenza in Minnesota. In Proceedings: The 53rd North Central Avian Disease Conference. Minneapolis, USA.

[11] Swayne DE, Suarez DL, Sims LD (2013). Influenza. In: Swayne DE, Glisson JR, Mc Dougald LR, et al. (Eds). Disease of poultry. 13th ed. Ames, USA: Wiley-Blackwell Inc..

[12] Eterradossi N, Saif YM (2013). Infectious bursal disease In: Swayne DE, Glisson JR, McDougald LR, et al. Disease of poultry. 13th ed. Ames, USA: Wiley-Blackwell Inc..

[13] Razmyar J, Peighambari SM (2009). Isolation and characterization of a very virulent Infectious bursal disease virus from turkey. Acta Virol.

[14] Owoade AA, Mulders MN, Kohnen J (2004). High sequence diversity in infectious bursal disease virus serotype 1 in poultry and turkey suggests West-African origin of very virulent strains. Arch Virol.

[15] Jackwood DJ, Stoute ST, Crossley BM (2016). Pathogenicity of genome reassortant infectious bursal disease viruses in chickens and Turkeys. Avian Dis.

[16] Reed LJ, Muench H (1938). A simple method of estimating fifty percent endpoint. Am J Hyg.

[17] Mayahi M, Bouzorghmehri Fard MH (2000). The effect of infectious bursal disease virus and cyclophosphamide on immune response against Newcastle disease vaccination in broiler chicks. Indian J Anim Sci.

[18] Azizpour A, Goudarzi H, Charkhkar S (2014). Experimental study on tissue tropism and dissemination of H9N2 avian influenza virus and ornithobacterium rhinotracheale coinfection in SPF chickens. J Anim Plant Sci.

[19] Lee CW, Suarez DL (2004). Application of real-time RT-PCR for the quantitation and competitive replication study of H5 and H7 subtype avian influenza virus. J Virol Methods.

[20] Dohms JE, Lee KP, Rosenberger JK (1981). Plasma cell changes in the gland of Harder infectious bursal disease virus infection of the chicken. Avian Dis.

[21] Edwards K, Logan J, Saunders N (2004). Real-time PCR: An essential guide.

[22] Swayne DE, Slemons RD (1994). Comparative pathology of a chicken origin and two duck origin influenza virus isolated in chickens, the effect of rout of inoculation. Vet Pathol..

[23] Mosleh N, Dadras H, Mohammadi A (2009). Molecular quantitation of H9N2 avian influenza virus in various organs of broiler chickens using TaqMan real time PCR. J Mol Genet Med..

[24] Swayne DE, Halvorson DA, Saif YM, Barnes HJ ( 2003). Influenza. Disease of poultry.

[25] Chui C, Thorsen J (1984). Experimental infection of turkeys with infectious bursal disease virus and the effect on the immunocompetence of infected turkeys. Avian Dis.

[26] Faragher JT, Allan WH, Wyeth CJ (1974). Immuno­suppressive effect of infectious bursal agent on vaccination against Newcastle disease. Vet Rec.

[27] Kwon JS, Lee HJ, Lee DH (2008). Immune responses and pathogenesis in immunocompromised chickens in response to infection with H9N2 low pathogenic avian influenza virus. Virus Res.

[28] Swayne DE, Slemons RD (1995). Comparative pathology of intravenously inoculated wild duck and turkey origin type A influenza virus in chickens. Avian Dis.

[29] Swayne DE, Slemons RD (1990). Renal pathology in specific-pathogen-free chickens with a water fowl-origin type A influenza virus. Avian Dis.

[30] Shalaby AA, Slemons RD, Swayne DE (1994). Pathological studies of A/Chicken/Alabama/7395/75(H4N8) influenza virus in specific pathogen free laying hens. Avian Dis.

[31] Swayne DE, Slemons RD (1992). Evaluation of the kidney as a potential site of avian influenza virus persistence in chickens. Avian Dis..

[32] Hablolvarid MH, Sohraby-Haghdost I, Pourbakhsh SA (2003). A study on histopathologic changes in chicken following intravenous inoculation with avian influenza virus A/Chicken/Iran/259/1998 (H9N2). Arch Razi Ins.

